# Benefits of multi-micronutrient nutritional formula combined with aerobic exercise on children with attention deficit hyperactivity disorder: improvements in symptoms, cognition, executive function, and sleep

**DOI:** 10.3389/fped.2025.1650588

**Published:** 2025-09-26

**Authors:** Yanjuan Ye, Yun Wang

**Affiliations:** Department of Pediatrics, WuXi No.2 People’s Hospital, Wuxi, Jiangsu, China

**Keywords:** ADHD, nutritional supplementation, aerobic exercise, cognitive function, executive function, sleep quality

## Abstract

**Objectives:**

Attention-Deficit/Hyperactivity Disorder (ADHD) affects approximately 5% of children globally and is characterized by symptoms of inattention, hyperactivity, and impulsivity. This study examines the impact of a multi-micronutrient nutritional formula combined with aerobic exercise on ADHD symptoms, cognitive function, specifically the domain of executive function, and sleep quality.

**Methods:**

A retrospective analysis was conducted on 220 children aged 6–12 years diagnosed with ADHD according to the American Psychiatric Association (APA) guidelines, who had been treated at WuXi No.2 People's Hospital from November 2022 to October 2024. Participants were divided into two groups: the Multi-Micronutrient Nutritional Formula (MNF) group (*n* = 111) who received a three-month dietary supplementation and a MNF combined with Aerobic Exercise (MNF-AE) group (*n* = 109) who received both the nutritional formula and daily aerobic exercise. Evaluations included the Conners Parent Symptom Questionnaire (PSQ), Stroop Test, Wisconsin Card Sorting Test (WCST), Alternate Uses Task, and the Children's Sleep Habits Questionnaire (CSHQ).

**Results:**

Post-treatment, the MNF-AE group exhibited significant improvements in ADHD symptoms, demonstrated by reduced PSQ scores (mean 30.75 ± 3.87) compared to the MNF group (32.42 ± 4.17; *P* = 0.002). The MNF-AE group also showed enhanced cognitive processing speed and selective attention, evidenced by faster Stroop Test times (Stroop Word: 23.03 ± 7.53 s vs. 25.86 ± 5.37 s; *P* = 0.002). Executive function improved significantly, as evidenced by higher total correct scores in the WCST (76.98 ± 10.74 vs. 73.75 ± 12.44; *P* = 0.041) and creativity (assessed by the Alternate Uses Task) improved in originality and fluency (all *P* < 0.05). Moreover, the MNF-AE group had better overall sleep quality as indicated by lower CSHQ scores (72.63 ± 6.87 vs. 74.95 ± 6.22; *P* = 0.009).

**Conclusions:**

Combining multi-micronutrient supplementation with aerobic exercise may offer potential benefits for managing ADHD symptoms, improving cognitive function, as well as enhancing sleep quality in children with ADHD. These findings suggest potential additive effects of this combined intervention.

## Introduction

Attention-Deficit/Hyperactivity Disorder (ADHD) is a prevalent neurodevelopmental disorder characterized by symptoms of inattention, hyperactivity, and impulsivity. Affecting approximately 5% of children worldwide (1), ADHD poses significant challenges not only to affected individuals but also to families, educators, and health care systems (2, 3). The etiology of ADHD is multifaceted, involving a combination of genetic, environmental, and neurobiological factors. This complexity necessitates diverse therapeutic approaches that target various dimensions of the disorder. Traditional management strategies primarily involve pharmacological interventions such as psychostimulants and non-stimulant medications (4–6). However, these treatments may not adequately address all aspects of ADHD, often prompting the exploration of alternative and complementary therapeutic options.

Emerging evidence suggests that nutritional and lifestyle modifications can play a crucial role in ADHD management, potentially addressing underlying physiological and cognitive deficits. However, as highlighted by Lange et al. (7), despite promising preliminary findings on the benefits of minerals and specific diets, there remains insufficient evidence to recommend the use of micronutrients or probiotics, underscoring the need for further comprehensive studies. Nutritional factors, including deficiencies in essential vitamins and minerals, have been implicated in the pathophysiology of ADHD. Micronutrients such as zinc, magnesium, iron, and B-complex vitamins are fundamental to neurotransmitter synthesis and brain function. For instance, zinc and magnesium are integral to dopamine regulation, a neurotransmitter critical for attention and impulse control (8, 9). Iron is essential for dopamine biosynthesis, with deficiencies linked to more severe inattentiveness, restlessness, and hyperactivity (10, 11). B vitamins further contribute to cognitive health by supporting neurotransmitter metabolism and reducing oxidative stress, which may ameliorate ADHD symptoms (12, 13).

Concurrent with nutritional considerations, physical exercise is gaining recognition as a beneficial adjunct therapy for ADHD. Regular physical activity has been associated with enhanced cognitive performance and bolstered brain health (14). Aerobic exercise, in particular, has demonstrated efficacy in improving attention and executive function in children with ADHD (15). These cognitive benefits have been hypothesized to stem from increased cerebral blood flow, upregulation of neurotrophins such as brain-derived neurotrophic factor (BDNF), and modifications in catecholaminergic neurotransmission (16). Collectively, these changes support synaptic plasticity, neurogenesis, and optimal neurotransmitter availability, which are crucial for attenuating ADHD symptoms such as inattention, hyperactivity, and impulsivity (17, 18).

The integration of multi-micronutrient formulations with aerobic exercise presents a promising multimodal approach to ADHD management. By concurrently addressing nutritional deficiencies and promoting physical fitness, this strategy offers the potential to enhance both neurological and physiological aspects of health. Previous research suggests that combined interventions may result in substantial improvements in attention, behavior, and cognitive functions (19). However, comprehensive investigations exploring these combined effects remain sparse, necessitating further research to elucidate the underlying mechanisms and optimize intervention protocols.

To address this gap, the present study evaluated the impact of a multi-micronutrient nutritional formula combined with aerobic exercise on key outcomes in children aged 6–12 years diagnosed with ADHD. This study aimed to comprehensively evaluate the benefits of a combined multi-micronutrient nutritional formula with aerobic exercise on symptom reduction, cognitive enhancement, improved executive function, and better sleep quality in children with ADHD.

## Methods

### Case selection: participant identification and grouping

#### Ethics statement: approval and consent waiver

The study received approval from the Institutional Review Board and Ethics Committee of WuXi No.2 People's Hospital. Since the research exclusively utilized de-identified patient data, posing no potential harm or impact on participants, the need for informed consent was waived. This waiver was granted in accordance with the regulatory and ethical standards applicable to retrospective research.

#### Study design and data collection: retrospective analysis approach

A retrospective analysis was performed on 220 children diagnosed with ADHD at WuXi No.2 People's Hospital between November 2022 and October 2024. The participants were categorized into two groups based on their treatment regimen. The first group, the Multi-Micronutrient Nutritional Formula (MNF) group, included 111 patients who received only the multi-micronutrient nutritional formula. The second group, the Multi-Micronutrient Nutritional Formula combined with Aerobic Exercise (MNF-AE) group, comprised of 109 patients who received both the multi-micronutrient nutritional formula and aerobic exercise therapy. Allocation to the MNF or MNF-AE group was based on the treatment regimen prescribed by the attending physician, ensuring that the allocation process was consistent with clinical practice and minimized potential selection bias. Eligible participants were identified from the hospital's medical records system, which contained comprehensive data on all ADHD patients treated during the specified period, encompassed demographic information, scores from the PSQ, WCST outcomes, performance on the Alternate Uses Task, and scores from the CSHQ.

The sample size for this study was calculated using GraphPad software. The calculation was based on the following assumptions: a medium effect size (Cohen's *d* = 0.5), a two-tailed significance level of *α* = 0.05, and a desired statistical power of 95%. It was estimated that each group required a minimum of 105 participants to detect a significant difference in mean scores using a two-sided, two-sample *t*-test with equal variances. This ensures adequate power to identify meaningful differences between the MNF and MNF-AE groups.

#### Inclusion and exclusion criteria: defining study eligibility

Participants were children diagnosed with ADHD, aged 6–12 years old. Specific inclusion and exclusion criteria are detailed below:

Inclusion criteria comprised the following: (1) a diagnosis of ADHD as per the American Psychiatric Association (APA) guidelines (20); (2) aged between 6 and 12 years (under age 6 years excluded due to potential challenges in their participation in the intervention; over 12 years excluded to avoid complications arising from complex developmental changes that could influence the study outcomes); and (3) availability of comprehensive medical records for each participant to facilitate thorough review and analysis.

The exclusion criteria were as follows: (1) the presence of exercise contraindications as per the American College of Sports Medicine (ACSM) guidelines (21), or any acute or chronic conditions potentially impacting the effectiveness of aerobic exercise intervention; (2) allergy to the micronutrient supplement administered in the study; (3) a diagnosis of other severe psychiatric or neurological disorders; (4) the presence of a severe physical illness or disability; (5) current use of asthma or allergic rhinitis medications; (6) the use of melatonin or similar substances for sleep enhancement; (7) participation in ongoing behavioral therapy or psychological treatment; and (8) intellectual disability or a history of brain injury.

### Intervening method: treatment regimens detailed: MNF and AE regimens

Both regimes were three-months in duration. The MNF group underwent a regimen involving a multi-micronutrient nutritional formula. This formula included essential micronutrients with adjusted dosages: Zinc (8 mg/day), Magnesium (150 mg/day), Iron (8 mg/day), Vitamin B1 (Thiamine, 0.9 mg/day), Vitamin B2 (Riboflavin, 1.1 mg/day), Vitamin B3 (Niacin, 12 mg/day), Vitamin B6 (Pyridoxine, 1.0 mg/day), Vitamin B12 (Cobalamin, 1.8 µg/day), and Folate (200 µg/day) These micronutrients were provided through high-quality supplements sourced from DSM Nutritional Products to ensure purity and potency. Additionally, participants received these nutrients through carefully monitored natural food sources such as red meat, seafood, legumes, whole grains, nuts, seeds, leafy green vegetables, and fortified foods. The supplements were administered in capsule form, ensuring ease of consumption and consistent dosage. The daily dietary intake was supervised by professional medical personnel, ensuring that all children consumed the same foods. No other dietary restrictions were imposed on the participants during the MNF regimen, except for adherence to the standardized multi-micronutrient formula.

The MNF-AE group combined the multi-micronutrient nutritional formula with aerobic exercise. Participants engaged in a daily aerobic exercise regimen consisting of a 5-min warm-up, 20 min of running, and a 5-min cool-down, totaling 30 min of exercise daily. This was conducted through outdoor running accompanied by one of the parents to ensure safety and support, while participants wore chest-belt heart rate monitors (V800, Polar Electro Oy, Finland) to track their heart rates. The exercise intensity was maintained at 50%–70% of each participant's Heart Rate Reserve (HRR), as recommended by the ACSM guidelines (21) for moderate intensity. The HRR was calculated as the difference between each participant's Maximal Heart Rate (MHR) and Resting Heart Rate (RHR).

This study employed a retrospective design, wherein data on both exercise adherence and nutritional supplement intake were collected post-intervention using structured interviews and medical records. All intervention protocols described herein were consistent with standard clinical practices during the original treatment period, ensuring methodological consistency and reliability.

### Data collection and outcome measurement: assessment tools utilized

#### ADHD symptoms

The Conners PSQ was employed to assess ADHD symptoms both before and after the intervention. It comprises a total of 48 items, each rated on a 4-point scale ranging from 0 to 3, resulting in a maximum possible score of 144. A higher score reflects more severe behavioral problems. The PSQ demonstrates a Cronbach's *α* of 0.86, indicating high reliability (22).

#### Stroop test

To assess attentional inhibitory control, participants completed the Stroop Color-Word Interference Test. In this test, participants were required to quickly and accurately verbally identify the ink color of displayed words. The Stroop Test included three conditions: Stroop Word, Stroop Color, and Stroop Color-Word. In the Stroop Word condition, participants completed 50 trials with color names printed in black ink. For the Stroop Color condition, there were 50 trials where color names appeared within rectangles matching the corresponding color. In the Stroop Color-Word condition, 50 trials consisted of color names printed in ink of a different color. Each condition's trials were arranged on a sheet of paper, with participants instructed to name the colors from top to bottom across 10 items per row and from left to right across 5 columns. The primary outcome measured in the Stroop Test was the time score, defined as the average time taken (in seconds) to correctly name the ink color for each trial. Lower time scores indicate better performance, reflecting more efficient attentional inhibitory control. The Stroop Color-Word Interference Test was selected because it has been shown to effectively measure executive function, particularly attentional inhibitory control. Additionally, this test was sensitive to acute exercise effects, making it suitable for evaluating changes in cognitive performance following interventions (23).

#### Wisconsin card sorting test

The WCST was employed to evaluate the executive function of participants. This test includes four stimulus cards and 128 response cards. Participants were instructed to sort the response cards based on one of three attributes—color, shape, or number—of the stimulus cards. After each attempt, the examiner provided feedback, indicating whether the match was “Correct” or “Incorrect”. Once a participant successfully matched 10 consecutive cards according to a specific attribute, termed the “nature of category”, the sorting rule was changed without prior notice. The test concluded either after the participant completed six categories or exhausted all 128 response cards.

For statistical analysis, six raw scores were recorded: Total Correct, Perseverative Responses, Perseverative Errors, Non-perseverative Errors, Conceptual Level Response, and Categories Completed. Perseverative Responses indicate the participant's tendency to continue sorting based on the previous category rule after it has changed. Perseverative Errors occur when the participant persists in following the old rule despite the change. Non-perseverative Errors were mistakes made while trying to adapt to the new rule. The Conceptual Level Response measures the ratio of correct sorts to total sorts within each category phase. Categories Completed refer to the number of category rules successfully mastered and completed by the participant. The WCST was chosen as one of the most widely used tasks for evaluating executive function. It was particularly effective in assessing cognitive flexibility, problem-solving abilities, and set-shifting (23).

#### Performance on the alternate uses task

To assess participants' cognitive flexibility and creativity, their performance on the “Alternate Uses Task” was evaluated. In this task, participants were required to generate as many alternative and novel uses as possible for a series of common household items. Each participant was given 60 s to provide verbal responses for each stimulus object, which were recorded with a dictation device placed before them. Prior to the task, an investigator trained in cognitive assessments delivered standardized instructions. During each session, participants completed one set of stimulus objects, consisting of three distinct items. Responses for each item were assessed based on four criteria: fluency, flexibility, originality, and elaboration. Elaboration scores ranged from 0 to 2 points, reflecting the depth and complexity of the ideas presented for each object. The Alternate Uses Task was included in our study because it has been found to be a valid and reliable cognitive test for assessing cognitive flexibility and creativity (24).

#### Sleep quality

The CSHQ was utilized to evaluate the children's sleep behaviors and disturbances. Parents rated their children's sleep conditions using a three-point scale: “frequently” (5–7 times per week), “sometimes” (2–4 times per week), and “rarely or never” (0–1 time per week). The CSHQ comprises 48 items, with 33 of these being scored and divided into 8 subscales. The total score can range from 33 to 99, with higher scores indicating poorer sleep quality. The CSHQ demonstrated a Cronbach's *α* coefficient of 0.73, indicating acceptable reliability (25).

### Statistical analysis: utilizing SPSS for data evaluation

Data analysis was conducted in SPSS 29.0 (SPSS Inc., Chicago, Illinois, USA). Continuous variables were assessed for normal distribution using the Shapiro–Wilk test. In cases where the continuous data followed a normal distribution, differences between groups were evaluated using the *T*-test. Categorical data were analyzed using the chi-square test. The test statistic is denoted as χ^2^. Throughout this study, any *P*-value below 0.05 was set as the threshold for statistical significance. Categorical data is presented as [*n* (%)], continuous data is presented as mean ± standard deviation (SD).

## Results

### Basic data

The mean age, gender distribution, ethnic distribution BMI, monthly household income per capita, distribution of ADHD subtypes, illness duration and average daily medication dosage showed no significant variation (*P* > 0.05). Thus, both groups were comparable (Table 1).

**Table 1:** Comparison of demographic characteristics between two groups.

Parameters	MNF group (*n* = 111)	MNF-AE group (*n* = 109)	*t*/*χ*^2^	*P*
Age (years)	9.42 ± 0.86	9.45 ± 0.94	0.251	0.802
Boys/Girls [*n* (%)]	61 (54.95%)/50 (45.05%)	53 (48.62%)/56 (51.38%)	0.883	0.347
Ethnicity (Han/Other) [*n* (%)]	106 (95.5%)/5 (4.5%)	102 (93.58%)/7 (6.42%)	0.392	0.531
BMI (kg/m^2^)	16.73 ± 2.54	16.74 ± 2.67	0.025	0.980
Monthly household income/person (2,000–5,000/5,000–10,000/above 10,000) [*n* (%)]	9 (17.12%)/64 (57.66%)/28 (25.23%)	15 (13.76%)/58 (53.21%)/36 (33.03%)	1.748	0.417
ADHD type [*n* (%)]				
-ADHD-I	37 (33.33%)	49 (44.95%)	3.472	0.176
-ADHD-HI	18 (16.22%)	12 (11.01%)		
-ADHD-C	56 (50.45%)	48 (44.04%)		
Duration of illness (year)	2.41 ± 1.26	2.35 ± 0.92	0.368	0.713
Daily medication (mg)	27.36 ± 9.11	26.78 ± 8.58	0.484	0.629

MNF, multi-micronutrient nutritional formula; MNF-AE, multi-micronutrient nutritional formula combined with aerobic exercise; BMI, body mass index; ADHD, attention deficit hyperactivity disorder; ADHD-I, predominantly inattentive subtype; ADHD-HI, predominantly hyperactive-impulsive subtype; ADHD-C, combined hyperactive-impulsive and inattentive subtype.

### ADHD symptoms

**Figure 1:**
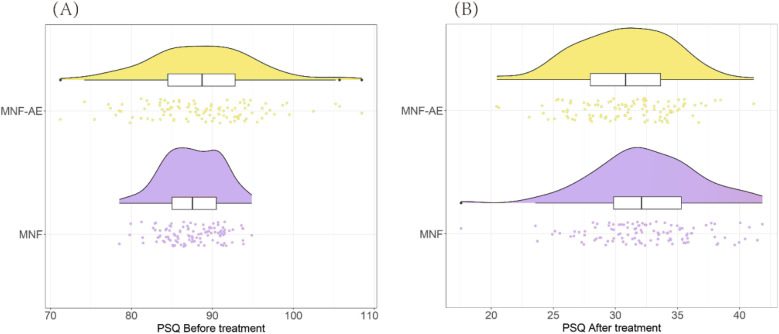
Comparison of PSQ score between two groups. **(A)** PSQ before treatment; **(B)** PSQ after treatment. ns: ns: no statistically significant difference; **: *P* < 0.01. PSQ: Conners Parent Symptom Questionnaire.

Prior to treatment, there was no difference in mean PSQ scores (*P* = 0.127) (Figure 1). However, after the intervention, the MNF-AE group exhibited significantly lower PSQ scores compared to the MNF group (*P* = 0.002).

### Attentional inhibitory control

Before treatment there was no difference in mean Stroop Word (*P* = 0.729), Stroop Color (*P* = 0.770), or Stroop Color-Word test times (*P* = 0.710) (Table 2).

**Table 2:** Comparison of Stroop test scores between two groups (before treatment).

Parameters	MNF group (*n* = 111)	MNF-AE group (*n* = 109)	*t*	*P*
Stroop Word (s)	26.54 ± 6.65	26.85 ± 6.44	0.347	0.729
Stroop Color (s)	36.27 ± 11.57	36.66 ± 7.93	0.293	0.770
Stroop Color-Word (s)	66.38 ± 17.63	67.23 ± 16.34	0.373	0.710

**Figure 2:**
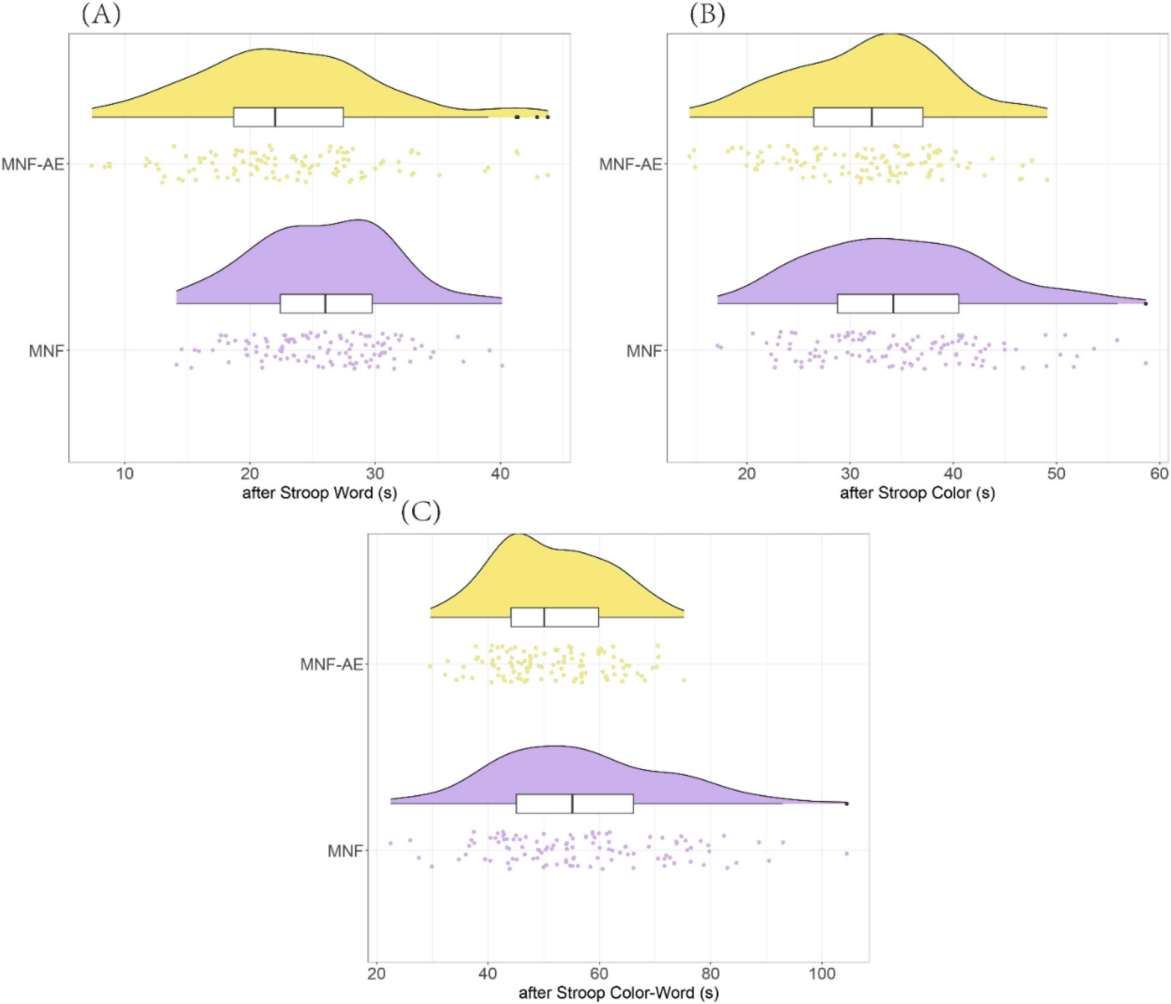
Comparison of stroop test results between two groups (after treatment). **(A)** Stroop Word; **(B)** Stroop Color; **(C)** Stroop Color-Word. **: *P* < 0.01.

After treatment, the MNF-AE group demonstrated significantly faster completion times compared to the MNF group in the Stroop Word (*P* = 0.002) the Stroop Color (*P* = 0.003), and Stroop Color-Word test (*P* = 0.002) (Figure 2).

### Executive function

Before treatment there was no difference between groups for total correct scores (*P* = 0.756), perseverative responses (*P* = 0.763), perseverative errors (*P* = 0.648), non-perseverative errors (*P* = 0.799), conceptual level response (*P* = 0.858), or categories completed (*P* = 0.465) (Table 3).

**Table 3:** Comparison of WCST results between two groups (before treatment).

Parameters	MNF group (*n* = 111)	MNF-AE group (*n* = 109)	*t*	*P*
Total correct (+)	68.87 ± 11.44	68.29 ± 15.82	0.311	0.756
Perseverative responses (–)	24.43 ± 8.87	24.78 ± 8.33	0.301	0.763
Perseverative errors (–)	20.74 ± 6.34	21.17 ± 7.54	0.457	0.648
Non-perseverative errors (–)	23.68 ± 6.88	23.92 ± 7.28	0.255	0.799
Conceptual level response (+)	55.36 ± 15.23	54.98 ± 16.43	0.179	0.858
Categories completed (+)	4.25 ± 1.27	4.38 ± 1.33	0.731	0.465

(+), higher scores represent better performance; (–), lower scores represent better performance.

**Figure 3:**
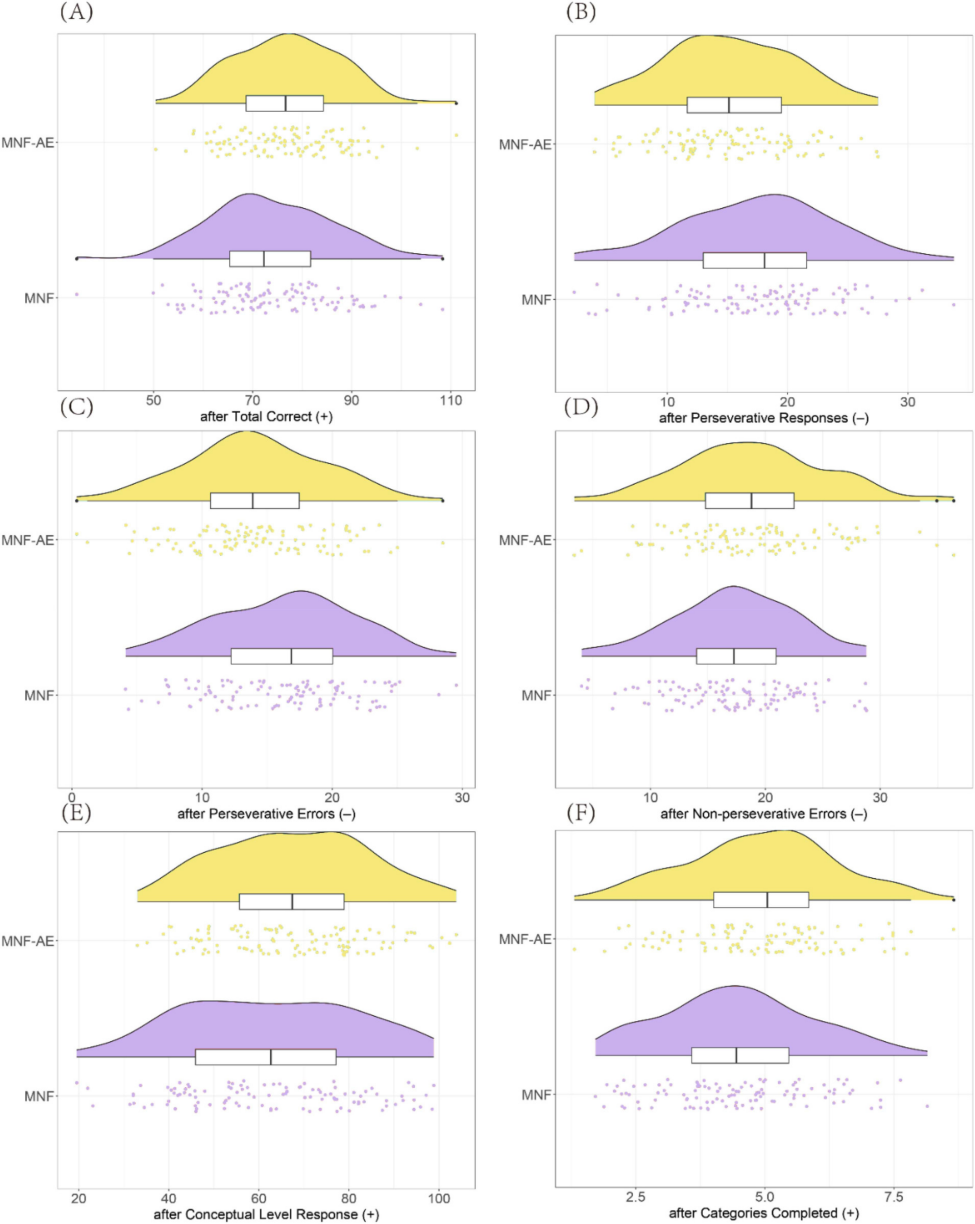
Comparison of WCST results between two groups (after treatment). **(A)** Total Correct; **(B)** Perseverative Responses; **(C)** Perseverative Errors; **(D)** Non-perseverative Errors; **(E)** Conceptual Level Response; **(F)** Categories Completed. (+): higher scores represent better performance; (–): lower scores represent better performance. *: *P* < 0.05; **: *P* < 0.01. WCST: Wisconsin Card Sorting Test.

After treatment, the MNF-AE group demonstrated a significantly higher total correct score (*P* = 0.041), significantly fewer perseverative responses (*P* = 0.002) and perseverative errors (*P* = 0.002), significantly higher conceptual level responses (*P* = 0.034), and completed more Categories (*P* = 0.020) compared to the MNF group. However non-perseverative errors were lower in the MNF group compared to the MNF-AE group (*P* = 0.036) (Figure 3).

### Cognitive function

The MNF-AE group outperformed the MNF group in scores for Category (*P* = 0.002), Fluency (*P* = 0.005), Originality (*P* = 0.013), and Elaboration (*P* = 0.033) (Table 4).

**Table 4:** Comparison of performance on the alternate uses task following aerobic exercise between two groups.

Parameters	MNF group (*n* = 111)	MNF-AE group (*n* = 109)	*t*	*P*
Category	3.49 ± 1.32	4.03 ± 1.25	3.117	0.002
Fluency	4.11 ± 1.54	4.77 ± 1.86	2.861	0.005
Originality	0.78 ± 0.22	0.87 ± 0.28	2.510	0.013
Elaboration	0.54 ± 0.16	0.59 ± 0.19	2.147	0.033

### Sleep parameters

Post-intervention, the MNF-AE group showed better improvement in bedtime resistance (*P* = 0.021), sleep onset delay (*P* = 0.013), and sleep anxiety (*P* = 0.020) compared to the MNF group (Table 5). No significant differences were observed in sleep duration (*P* = 0.222), sleep-disordered breathing (*P* = 0.361), parasomnias (*P* = 0.503), daytime sleepiness (*P* = 0.279), or night wakings (*P* = 0.124). Additionally, total CSHQ scores were significantly lower in the MNF-AE group (*P* = 0.009). The effect sizes for various parameters were calculated and are summarized in Table 6.

**Table 5:** Comparison of CSHQ score following aerobic exercise between two groups.

Parameters	MNF group (*n* = 111)	MNF-AE group (*n* = 109)	*t*	*P*
Bedtime resistance	14.74 ± 1.34	14.26 ± 1.66	2.334	0.021
Sleep onset delay	11.36 ± 1.63	10.86 ± 1.32	2.500	0.013
Sleep anxiety	8.23 ± 1.77	7.69 ± 1.65	2.336	0.020
Sleep duration	7.15 ± 1.34	6.93 ± 1.33	1.225	0.222
Sleep disordered breathing	5.14 ± 1.58	4.95 ± 1.62	0.916	0.361
Parasomnias	8.24 ± 1.63	8.11 ± 1.23	0.671	0.503
Daytime sleepiness	13.98 ± 1.33	13.74 ± 1.85	1.086	0.279
Night wakings	5.94 ± 1.63	6.24 ± 1.26	1.546	0.124
Total	74.95 ± 6.22	72.63 ± 6.87	2.621	0.009

**Table 6:** Effect size.

Parameters	Cohen_d	Parameters	Cohen_d
Age (years)	0.034	After perseverative responses (–)	0.421
BMI (kg/m^2^)	0.003	After perseverative errors (–)	0.430
Duration of illness (year)	0.049	After non-perseverative errors (–)	0.285
Daily medication (mg)	0.065	After conceptual level response (+)	0.288
PSQ before treatment	0.208	After categories completed (+)	0.315
PSQ after treatment	0.414	Category	0.420
Before stroop word (s)	0.047	Fluency	0.386
Before stroop color (s)	0.039	Originality	0.339
Before stroop color-Word (s)	0.050	Elaboration	0.289
After stroop word (s)	0.434	Bedtime resistance	0.315
After stroop color (s)	0.403	Sleep onset delay	0.336
After stroop color-word (s)	0.423	Sleep anxiety	0.315
Before total correct (+)	0.042	Sleep duration	0.165
Before perseverative responses (–)	0.041	Sleep disordered breathing	0.124
Before perseverative errors (–)	0.062	Parasomnias	0.090
Before non-perseverative errors (–)	0.034	Daytime sleepiness	0.147
Before conceptual level response (+)	0.024	Night wakings	0.208
Before categories completed (+)	0.099	Total	0.353
After total correct (+)	0.278		

## Discussion

The major finding of the present study was that the addition of aerobic exercise into a regimen of multi-micronutrient supplementation significantly improved cognition, sleep, and ADHD symptomology in children with ADHD. Our findings indicate that integrating aerobic exercise into a regimen of multi-micronutrient supplementation improved PSQ scores in children with ADHD. This improvement can be attributed to the combined effects of nutrient support, which addresses deficiencies critical for neurotransmitter synthesis and neural function, and exercise-induced neuroplasticity, promoting brain regions involved in executive function and attention (26, 27). Specifically, the MNF-AE group likely benefited from better regulation of dopamine and norepinephrine due to nutrient intake (28), and enhanced synaptic plasticity and neurogenesis from aerobic exercise (29), leading to improved ADHD symptom management as reflected in the PSQ scores. These findings are consistent with Zhu et al.'s (30) systematic review and network meta-analysis, which found that closed-skill activities dominated by aerobic exercises were particularly effective in improving hyperactivity/impulsivity and inattentionin children and adolescents with ADHD.

It appears that the incorporation of aerobic exercise provides a complementary mechanism for improving sleep, cognition, and symptomology in children with ADHD, potentially operating through enhanced cerebral blood flow and the upregulation of neurotrophins like BDNF. Exercise-induced BDNF formation was known to promote synaptic plasticity and neurogenesis, potentially leading to improved cognitive outcomes, such as those demonstrated by Jaberi et al. (31), who found that physical exercise increases BDNF levels, enhancing learning and memory. The present study observed improvements in cognitive performance (better performance on the Stroop Test and WCST) when aerobic exercise was used in addition to a MNF which adds evidence for the use of aerobic exercise to improve attentional control and executive functioning. This aligns with previous research showing that regular physical activity can lead to structural changes in the brain, particularly in areas associated with executive function such as the prefrontal cortex and hippocampus (17, 18, 32, 33). However, it is important to consider other perspectives. For instance, an umbrella review by Dastamooz found strong evidence for the effectiveness of exercise in improving inattention and inhibitory control but noted weaker evidence for emotional and social outcomes (34). Therefore, while our findings support the cognitive benefits of aerobic exercise, future research should further explore its impact on these less robust outcomes.

A proposed mechanism for this improvement in attentional control and executive functioning with the addition of aerobic exercise is the ability of aerobic exercise to modulate catecholaminergic activity. Specifically, dopamine and norepinephrine, which are crucial in regulating attention and impulse control. Exercise-induced increases in these neurotransmitters' availability are suggested to optimize arousal mechanisms and enhance cognitive clarity (35, 36), which may explain the improvements in improved cognitive processing speed and selective attention with the addition of aerobic exercise observed in the present study.

The improvement in executive function indices, such as reduced perseverative errors and increased total correct scores in the WCST observed for the MNF-AE group in the present study, highlights the potential for physical activity to influence cognitive flexibility. Evidence from prior literature suggests that these improvements may be mediated by the exercise-induced enhancement of prefrontal cortex function, which is critical for executive task performance (37). This may be of specific benefit to children with ADHD for whom responses to changing contingencies is a common challenge as improved problem-solving skills facilitate adaptive responses to changing contingencies (24).

The Alternate Uses Task results suggest that the combined intervention positively affects divergent thinking through changes in neural pathways that promote novel idea generation. This process aligns with reports of dynamic changes in functional connectivity post-exercise, fostering a cognitive environment conducive to creative thinking (38, 39).

Additionally, sleep quality, assessed via the CSHQ, showed marked improvement in the MNF-AE group. While significant enhancements were observed in specific aspects of sleep, it is important to acknowledge that not all sleep parameters demonstrated improvements. The combination of aerobic exercise and micronutrient supplementation appears to be particularly effective in enhancing sleep onset, reducing bedtime resistance, and alleviating sleep-related anxiety. A critical factor here was that both exercise and micronutrients may exert beneficial effects on the sleep-wake cycle, more so when systematically combined (40, 41). Exercise generally promotes better sleep through thermoregulatory and circadian rhythm influences, while adequate nutrient intake ensures the presence of precursors required for sleep modulating neurotransmitters such as serotonin and melatonin (42, 43). These effects likely contributed to better sleep onset, reduced anxiety, and overall improved sleep quality in the MNF-AE group, offering the additional benefit of potentially mitigating the negative impact of poor sleep on ADHD symptoms (44, 45).

Interestingly, our study also suggests cultural and behavioral elements associated with dietary habits and physical activity can significantly influence health outcomes in children with ADHD. The structured regimen likely promoted a consistent daily routine, facilitating adherence, which were particularly beneficial in managing ADHD. Furthermore, the consistency in exercise and diet may instill discipline, indirectly benefiting behavioral management (46–48).

The combination of multi-micronutrient supplementation (MNF) and aerobic exercise offers a promising approach to managing ADHD symptoms in children. By addressing nutritional deficiencies, MNF ensures that essential vitamins and minerals are available for optimal neurotransmitter function, which supports better regulation of critical neurotransmitters like dopamine and norepinephrine (7, 49). Aerobic exercise complements this by enhancing cerebral blood flow and promoting neurotrophin upregulation, such as BDNF, which fosters synaptic plasticity and neurogenesis (50). Together, these interventions create an environment conducive to improved ADHD behavioral management (15). In real-world settings, this combined approach can be particularly beneficial for children with ADHD. It not only helps manage core symptoms but also establishes a structured routine that aids in maintaining consistency and discipline. For example, the integration of regular physical activity and balanced nutrition can help children develop healthier habits, leading to long-term benefits in their daily functioning and overall quality of life. These findings suggest that combining nutritional and physical activity interventions could provide a holistic and non-pharmacological strategy for ADHD management, potentially reducing reliance on traditional treatments and improving outcomes for affected children.

While this study provides promising insights into the effects of multi-micronutrient supplementation combined with aerobic exercise in managing ADHD in children, it was not without limitations. The sample size was relatively small, which may limit the generalizability of the findings to broader populations. The reliance on parent-reported measures also introduces potential bias. Objective sleep measurements, such as actigraphy or polysomnography, would provide more reliable data and should be included in future research to better understand the impact of the intervention on sleep. Future research with larger, more diverse populations and a comprehensive assessment of confounding variables was necessary to validate these findings and optimize the intervention protocol. Furthermore, the retrospective nature of this study meant that treatments were not randomized or prospectively assigned. This design limitation might have introduced biases and confounding factors that could affect the validity of our findings. The study lacked both a control group receiving usual treatment and an exercise-only group, making it difficult to attribute observed improvements directly to the intervention. Another limitation is that the caloric value of the food was not adjusted to account for the higher energy expenditure in the MNF-AE group due to daily aerobic exercise. This means that the energy balance may have differed between groups, potentially affecting the comparability of the MNF intervention. Future research should address this limitation by ensuring that caloric intake is adjusted to match energy expenditure across different intervention groups, thereby providing more reliable comparisons. Our statistical analysis did not adjust for potential confounders, such as baseline cognitive function, parental education level, and comorbid conditions, which could have influenced the outcomes. This omission is particularly critical because it may lead to biased estimates of the intervention's effectiveness. Additionally, it did not explore interaction effects between time and intervention. This limitation is especially important when considering outcomes susceptible to learning effects, such as performance on the Stroop test. Recognizing these limitations, we advocate for future studies employing prospective, randomized designs with comprehensive statistical adjustments to better understand the effects of multi-micronutrient supplementation combined with aerobic exercise on ADHD symptoms in children.

## Conclusion

In conclusion, while traditional treatments for ADHD focus on symptom management, the addition of a multi-micronutrient nutritional formula combined with aerobic exercise may offer potential benefits as part of a multimodal approach. This strategy not only addresses underlying nutritional deficiencies and enhances physical health but also supports cognitive and neurochemical processes linked to ADHD. These results underscore the potential value of considering holistic interventions that encompass dietary and lifestyle modifications alongside conventional therapies. Future research should explore long-term effects, optimal intervention durations, and potential biochemical markers that can further elucidate the mechanisms driving these benefits, paving the way for more personalized and integrative ADHD management strategies.

## Data Availability

The raw data supporting the conclusions of this article will be made available by the authors, without undue reservation.
